# Prospective clinical and radiographic evaluation of an allogeneic bone matrix containing stem cells (Trinity Evolution® Viable Cellular Bone Matrix) in patients undergoing two-level anterior cervical discectomy and fusion

**DOI:** 10.1186/s13018-017-0564-5

**Published:** 2017-04-26

**Authors:** Timothy A. Peppers, Dennis E. Bullard, Jed S. Vanichkachorn, Scott K. Stanley, Paul M. Arnold, Erik I. Waldorff, Rebekah Hahn, Brent L. Atkinson, James T. Ryaby, Raymond J. Linovitz

**Affiliations:** 1 Seaside Spine Medical Associates, 320 Santa Fe Dr., Suite 300, Encinitas, CA 92024 USA; 2 Triangle Neurosurgery, 1540 Sunday Dr., Raleigh, NC 27607 USA; 3 Tuckahoe Orthopaedic Associates, 1501 Maple Ave., Richmond, VA 23226 USA; 4 Denver-Vail Orthopedics, P.C., 8101 E. Lowry Blvd., Suite 260, Denver, CO 80230 USA; 5 Kansas University Medical Center, 3901 Rainbow Blvd Ste 2B, Kansas City, KS 66160 USA; 6 Orthofix, Inc., 3451 Plano Parkway, Lewisville, TX 75056 USA; 7 Atkinson Biologics Consulting, Highlands Ranch, CO 80129 USA; 8 PO Box 1671, Rancho Santa Fe, CA 92067 USA

**Keywords:** ACDF, PEEK cage, Allograft, Multilevel, Arthrodesis, Cervical spine, Spine fusion

## Abstract

**Background:**

Trinity Evolution® (TE), a viable cellular bone allograft, previously demonstrated high fusion rates and no safety-related concerns after single-level anterior cervical discectomy and fusion (ACDF) procedures. This prospective multicenter clinical study was performed to assess the radiographic and clinical outcomes of TE in subjects undergoing two-level ACDF procedures.

**Methods:**

In a prospective, multicenter study, 40 subjects that presented with symptomatic cervical degeneration at two adjacent vertebral levels underwent instrumented ACDF using TE autograft substitute in a polyetherethereketone (PEEK) cage. At 12 months, radiographic fusion status was evaluated by dynamic motion plain radiographs and thin cut CT with multiplanar reconstruction by a panel that was blinded to clinical outcome. Fusion success was defined by angular motion (≤4°) and the presence of bridging bone across the adjacent vertebral endplates. Clinical pain and function assessments included the Neck Disability Index (NDI), neck and arm pain as evaluated by visual analog scales (VAS), and SF-36 at both 6 and 12 months.

**Results:**

At both 6 and 12 months, all clinical outcome scores (SF-36, NDI, and VAS pain) improved significantly (*p* < 0.05) compared to baseline values. There were no adverse events or infections that were attributed to the graft material, no subjects that required revisions, and no significant decreases to mean neurological evaluations at any time as compared to baseline. At 12 months, the per subject and per level fusion rate was 89.4 and 93.4%, respectively. Subgroup analysis of subjects with risk factors for pseudoarthrosis (current or former smokers, diabetic, or obese/extremely obese) compared to those without risk factors demonstrated no significant differences in fusion rates.

**Conclusions:**

Patients undergoing two-level ACDF with TE in combination with a PEEK interbody spacer and supplemental anterior fixation had a high rate of fusion success without any serious adverse events related to the graft material.

**Trial registration:**

Trinity Evolution in Anterior Cervical Disectomy and Fusion (ACDF) NCT00951938

## Background

Symptomatic cervical disc degeneration includes a multitude of pathologic processes including decreased disc height, disc herniation, and spondylosis resulting in radiculopathy and/or myelopathy. Anterior cervical discectomy and fusion (ACDF) is an established surgical treatment that achieves good to excellent clinical results in patients with symptomatic cervical degenerative disc disease [1]. Although multilevel ACDF is a safe and reliable procedure, multilevel procedures are associated with an increased rate of reoperation, higher non-union rates and longer time to fusion as compared to single-level procedures [2–6]. Additionally, patients who use tobacco [7] and particularly smokers who had a 2-level ACDF [8] have been associated with increased rates of pseudoarthrosis.

To minimize this risk of pseudoarthrosis, surgeons may select from a variety of bone graft materials with various qualities. Few bone graft substitutes contain all three essential bone-forming elements of autograft (osteogenicity, osteoconductivity, and osteoinductivity) [9] in a single, off-the-shelf product. Trinity Evolution**®** (TE) is a cellular bone allograft that consists of viable cellular cancellous bone matrix and demineralized cortical bone. TE possesses all three essential elements that are required for successful bone grafting, physiologic numbers of osteogenic cells (including mesenchymal stem cells and osteoprogenitor cells), osteoinductive proteins, and an osteoconductive matrix to which the cells are attached [10]. In a prospective study that evaluated the safety and effectiveness of TE in single-level ACDF, the fusion rate was 93.5% at 12 months, no serious allograft-related events occurred and comparisons to the literature revealed that TE may help negate any comorbid physiological barriers to fusion associated with risk factors such as smoking and diabetes [11].

The primary aim of this multicenter clinical study was to prospectively assess the safety and effectiveness of the TE viable cellular bone allograft in combination with a polyetherethereketone (PEEK) interbody spacer in two-level ACDF using patient reported and radiological outcome measures. To better assess effectiveness, the fusion rates were compared with the international literature that described a comparable surgical approach using other graft materials. A secondary aim of the study was to compare fusion rates between patients with and without risk factors for pseudoarthrosis.

## Methods

### Study design

From October 2009 to June 2012, a prospective, multicenter study was conducted at five investigational sites to evaluate the safety and effectiveness of a cellular bone allograft (Trinity Evolution® (TE)) in combination with a PEEK interbody spacer for ACDF surgery. All patients 18 years of age or older with symptomatic cervical degeneration at two adjacent vertebral levels between C3 and T1 were eligible for the study and those enrolled underwent ACDF with supplemental fixation and a PEEK interbody spacer (Orthofix, Inc., Lewisville, TX). TE was packed within and around the spacer. Exclusion criteria included the use of any other bone graft or bone graft substitute in addition to or in place of TE in and around the interbody spacer or arthrodesis at a single level only or at more than two levels. IRB approval was obtained for each site prior to the initiation of enrollment.

### Surgical procedures

All operations were performed by five surgeons using comparable surgical techniques. A standard Smith-Robinson approach to the cervical spine was carried out through a transverse incision. After removal of disc material and endplate cartilage, subchondral bone was perforated and the neural structures were decompressed. During distraction, a PEEK cage (Orthofix Inc. Lewisville, TX) packed with TE was inserted into the intervertebral space. Additionally, TE was packed around the cage if space permitted. Rigid anterior plate-screw fixation was performed in all patients.

### Postoperative management and data collection

Subjects were discharged from the hospital on the day of surgery or the day after surgery and were treated with comparable postoperative protocols. All subjects were allowed to ambulate on the first day after surgery. Post-operative immobilization in a cervical collar or brace was prescribed at the surgeon’s discretion.

Information regarding subject age, gender, body mass index (BMI), smoking status and the presence or absence of diabetes was collected. Subjects were evaluated clinically and radiographically at 6 (+/−1) weeks, 6 months (+/−1) and 12 (+/−1) months. At all timepoints, plain radiographs (flexion/extension, AP and lateral) and neurological evaluations (motor, sensory, or reflex) were collected. Neurologic evaluations included motor assessments of elbow flexors, wrist extensors, elbow extensors and finger extensions using a 0–5 scoring system. For sensory function, each cervical segment was assessed for absence, impaired, or normal function. For reflex assessment, biceps, brachioradialis, and triceps were evaluated using a four-point scale. Thin cut (≤1 mm) computed tomography with multiplanar reformatting (CT) was also performed for every subject at 12 months according to the study protocol.

Clinical endpoints included three health measurement instruments: the Neck Disability Index (NDI), visual analogue scale (VAS) (neck and arm), and the SF-36v2, which evaluated pain, function and quality of life (QOL). The NDI ranged from 0–50 points with higher scores representing greater functional improvement. The VAS scale ranged from 0 to 100 mm with 0 representing no pain and 100 representing severe pain on activity. The SF-36, an eight-scale profile of functional health and well-being scores, was summarized to obtain the physical composite score (PCS) and the mental composite score (MCS). In contrast to NDI and VAS, higher scores for SF-36 represent less disability.

### Radiographic evaluation

At 12 months, the criteria for fusion required the presence of bridging bone across the adjacent endplates on thin cut CT scans with multiplanar reformatting and ≤4° angular motion on flexion/extension plain radiographs. Both levels were required to be fused in order for the subject to be judged as fused. Radiographic fusion status was determined via an independent review by three qualified reviewers who possessed substantial orthopedic experience and either an MD or a PhD. All three reviewers had to independently agree that bridging bone was present in order for the site to be judged as fused. All radiographic evaluations were performed by reviewers blinded to the patient’s clinical outcomes. At 6 months, fusion was assessed by bony bridging based on plain radiographs.

The quantitative assessments of intervertebral motion were produced by trained analysts using specialized motion analysis software, QMA™ (Quantitative Motion Analysis; Medical Metrics, Inc., Houston, TX). QMA™ has been validated to produce measurements of intervertebral rotation and translation and is accurate to within 1 degree and 1 mm [12, 13]. The reproducibility of the measurements has also been validated [12, 13].

### International literature search

The literature search was conducted using PubMed with search terms for ACDF, PEEK, and two- or multilevel. Publications that were included must have reported a two-level ACDF procedure using a PEEK cage with supplemental fixation, the specific graft material, the follow-up times that fusion was assessed and the fusion incidence. Publications that were excluded were reports that described one-, three-, and four- level ACDF procedures or two-level ACDF reports that utilized autograft or allograft interbody spacers, a PEEK cage without graft material or a PEEK cage without rigid supplemental fixation (e.g., “stand-alone”).

### Statistical methods

The data in the figures and the results are presented as the mean and standard error (SE) and mean and standard deviation (SD), respectively. A multiple paired *t* test with a subsequent Bonferroni correction was done for subject reported outcome measures. The Fisher’s exact test was used to compare fusion rates among subjects with risk factors for pseudoarthrosis. Significance was set at *p* ≤ 0.05. The statistical analyses were performed using SAS (version 9.3, Cary, NC).

## Results

Forty subjects were enrolled in the study and arthrodesis was performed on 80 levels. Thirty-five and 38 subjects completed their 6 and 12 month study visits, respectively.

### Baseline characteristics

The mean age and standard deviation was 48.5 +/−9 years and the age range was 26–65 years of age. Demographics are described in Table 1. Twenty-six (65.0%), thirteen (32.5%), and one (2.5%) subject received arthrodesis at C5-C7, C4-C6, and C3-C5, respectively.

**Table 1:** Patient demographics

Patient demographic	*N* (%)
Gender	
Male	11 (27.5)
Female	29 (72.5)
Age	
<50 years	20 (50.0)
<65 years	38 (95.0)
Smoking status	
Never	22 (55.0)
Current or former	18 (45.0)
Diabetic	
No	35 (87.5)
Yes	5 (12.5)
Weight status (base on BMI)	
Normal weight	11 (27.5)
Overweight	9 (22.5)
Obese	16 (40.0)
Extremely obese	4 (10.0)

### Fusion

**Fig. 1:**
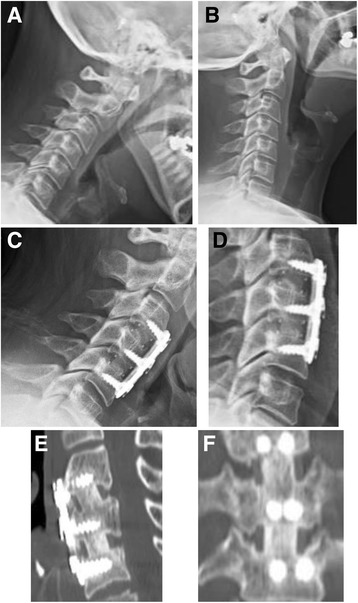
Two-level ACDF using Trinity Evolution that was performed on a 44-year-old obese female at C3-4 and C4-5. **a** Pre-operative flexion radiograph. **b** Pre-operative extension radiograph. **c** Twelve month flexion radiograph. **d** Twelve month extension radiograph. **e** Twelve month sagittal CT. **f** Twelve month coronal CT

The per subject fusion rate increased over time and was determined to be 65.7% of subjects fused at 6 months and 89.4% at 12 months (Table 2; Fig. 1). The per level fusion rate mirrored the increase over time that was observed in the per subject fusion rate and was 54.3 and 93.4% at 6 and 12 months, respectively (Table 2; Fig. 1). The fusion rates at 12 months for subjects that were current or former smokers, diabetic, or obese were 94.1% (16/17), 100% (5/5), and 93.3% (14/15), respectively. Subgroup analysis of these high risk subjects compared to subjects without risk factors demonstrated no significant differences (*p* > 0.05) in fusion rates at 12 months (not shown).

**Table 2:** Fusion rates at 6 and 12 months

	Per subject fusion		Per level fusion	
Time (M)	6	12	6	12
Fused *N* (%)	23 (65.7)	34 (89.4)	38 (54.3)	71 (93.4)
Not fused *N* (%)	12 (34.3)	4 (10.6)	32 (45.7)	5 (6.6)

### Clinical findings

**Fig. 2:**
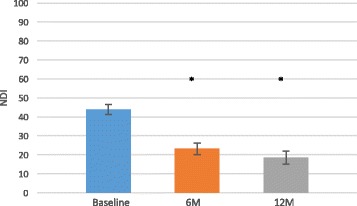
Neck Disability Index (NDI) mean scores improved over time. Data are presented as the score mean ± the standard error. An *asterisk* indicates that the NDI score at each individual postoperative time point demonstrated significantly (*p* < 0.0001) improved function scores as compared to baseline

**Fig. 3:**
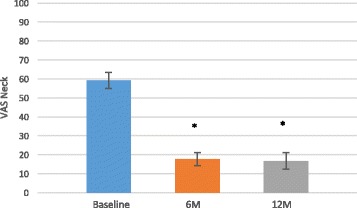
VAS neck mean pain scores improved over time. Data are presented as the score mean ± the standard error. An *asterisk* indicates that the VAS neck pain score at each individual postoperative time point demonstrated significantly (*p* < 0.0001) improved function scores as compared to baseline

**Fig. 4:**
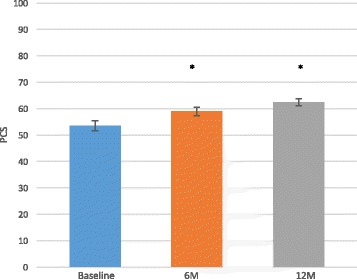
SF-36 PCS mean improvements over time. Data are presented as the score mean ± the standard error. The mean SF-36 PCS at 6 and 12 months demonstrated significantly (*p* < 0.05) improved function scores as compared to baseline

All patient reported outcomes (NDI, VAS neck and arm pain, SF-36 MCS and PCS) demonstrated significant improvements in pain and function at 6 and 12 months as compared to baseline (Figs. 2, 3, and 4).

### Safety

There were no adverse events or infections that were related to TE and no pseudoarthroses that required revisions. There was no neurological deterioration encountered (motor, sensory, or reflex) at any time as compared to baseline.

## Discussion

The primary aim of this multicenter, open-label clinical study was to prospectively assess the safety and effectiveness of Trinity Evolution cellular bone allograft (TE) in two-level ACDF procedures using a PEEK interbody cage and supplemental fixation, which is the standard of care for each of our five practices. The use of TE did not raise any safety concerns, since there were no adverse events, infections, or reoperations. All measures of subject pain and function (NDI, VAS neck and arm, SF-36 overall and MCS and PCS subscales) significantly improved at both 6 and 12 months as compared to baseline.

One secondary aim of the study was to compare the fusion rates of groups at risk of pseudoarthrosis with normal controls. Smokers [7] particularly smokers who had a two-level ACDF [8] have been associated with increased rates of pseudoarthrosis. Although the sample size was small, there were no significant differences observed between normal and at risk subjects. TE may help overcome the biological factors that impede healing in these groups, but this evaluation was underpowered and a clinically applicable conclusion cannot be drawn.

Because surgeons have several bone graft materials available, a literature review was performed to compare these fusion results to studies that used a comparable approach and instrumentation (Table 3). Evaluation of both safety and effectiveness can help surgeons select a preferred bone graft among the several types including cellular bone allograft, non-cellular allograft such as demineralized bone matrix (DBM), recombinant BMP containing grafts such as INFUSE®, and autograft. Since TE is a cellular bone allograft that contains DBM, one way to assess the potential benefit of TE is to compare the fusion incidence to studies that used DBM. The Topuz et al. study [14] demonstrated a 69.6% fusion rate using DBM, which is twenty percentage points lower than the 89.4% fusion rate for TE. Another study used DBM in conjunction with a synthetic graft material [15], which is a potential confounding factor for accurate data comparison. The use INFUSE® was described in ACDF procedures [16–18]. Although the fusion outcomes using INFUSE are high, there is a substantial safety issue when using INFUSE for ACDF procedures. FDA issued a public health notification of life-threatening cervical swelling (https://wayback.archive-it.org/7993/20170111190511/ http://www.fda.gov/MedicalDevices/Safety/AlertsandNotices/PublicHealthNotifications/ucm062000.htm) when INFUSE is used in the cervical spine. Table 3 also shows high fusion rate when autograft is used [15]. However, harvesting of autograft requires a second operative site which is associated with pain and morbidity that includes chronic harvest site pain, infection, increased operative time, and blood loss [19–23]. Thus, the results described herein appear promising because TE has the potential of increased arthrodesis rates as compared to allograft and TE lacks the safety concerns associated with INFUSE and autograft harvest.

**Table 3:** Literature describing fusion rates after a two-level ACDF procedure using a PEEK cage and supplemental fixation

Reference	*n*	Graft	Follow-up time (months)	Fusion rate (%)
Xie, 2015 [15]	19	CaS/DBM	12	94.3
	20	Autograft	12	100
Tumialan, 2008 [16]	62	INFUSE	8–36	100
Boakye, 2005 [17]	9	INFUSE	12–16	100
Lovasik, 2016 [18]	34	INFUSE	12	100
Topuz, 2009 [14]	79	DBM	12	69.6

Limitations to this study include a lack of a control group and thus TE treatment was not directly compared to autograft or non-cellular allograft treatments. Additionally, since the surgeons were not restricted with their use of operative approaches or fixation, either or both may have impacted outcomes. The impact of these factors on the outcome was not evaluated. Lastly, there was no sample size estimation in the protocol because there were no formal statistical hypotheses.

## Conclusions

In conclusion, subjects who received Trinity Evolution in combination with a PEEK interbody device during a two-level ACDF procedure had a high rate of fusion success both overall and when stratified into high-risk groups, while having no serious adverse events related to the graft material.

## Abbreviations

BMI: Body mass index
CaS: Calcium Sulfate
CT: Computed tomography
DBM: Demineralized bone matrix
IRB: Internal review board
MCS: Mental component score
PCS: Physical component score
PEEK: Polyetherethereketone
QMA: Quantitative motion assessment
SD: Standard deviation
SE: Standard error
SF-36: Short form 36
TE: Trinity Evolution
VAS: Visual analogue scale
